# Street Food Stand Availability, Density, and Distribution Across Income Levels in Mexico City

**DOI:** 10.3390/ijerph18083953

**Published:** 2021-04-09

**Authors:** Jose B. Rosales Chavez, Meg Bruening, Punam Ohri-Vachaspati, Rebecca E. Lee, Megan Jehn

**Affiliations:** 1 School of Geographical Sciences and Urban Planning, Arizona State University, 975 S. Myrtle Ave, Tempe, AZ 85281, USA; 2 College of Health Solutions, Arizona State University, 550 N. 3rd Street, Phoenix, AZ 85004, USA; meg.bruening@asu.edu (M.B.); punam.ohri-vachaspati@asu.edu (P.O.-V.); 3 Center for Health Promotion and Disease Prevention, Edson College of Nursing & Health Innovation, Arizona State University, 502 N. 3rd Street, Phoenix, AZ 85004, USA; releephd@yahoo.com; 4 School of Human Evolution and Social Change, Arizona State University, Tempe, AZ 85281, USA; megan.jehn@asu.edu

**Keywords:** food environment, food retail, street food stands, ground-truthing, geographic information systems, Mexico

## Abstract

Street food stands (SFS) are an understudied element of the food environment. Previous SFS studies have not used a rigorous approach to document the availability, density, and distribution of SFS across neighborhood income levels and points of access in Mexico City. A random sample (*n* = 761) of street segments representing 20 low-, middle-, and high-income neighborhoods were assessed using geographic information system (GIS) and ground-truthing methods. All three income levels contained SFS. However, SFS availability and density were higher in middle-income neighborhoods. The distribution of SFS showed that SFS were most often found near homes, transportation centers, and worksites. SFS availability near schools may have been limited by local school policies. Additional studies are needed to further document relationships between SFS availability, density, and distribution, and current structures and processes.

## 1. Introduction

The food environment can significantly impact individuals’ eating behaviors. It is an important factor to consider when addressing diet-related problems, such as overweight, obesity, and diabetes. The types of foods available in a community and the quality of those foods have been shown to be correlated with consumer health status [1,2,3,4]. Food environments with a high prevalence of fast-food restaurants and convenience stores are associated with adverse health outcomes, such as cardiovascular diseases, diabetes, and some types of cancer [4,5,6,7]. In contrast, food environments with ample supermarkets and grocery stores are associated with reduced risks for these same negative health outcomes [4,7].

Studies of food environments can help us understand the variations in numbers, locations, and types of food venues across communities. A community’s socioeconomic characteristics can explain some of these variations. For example, supermarkets and grocery stores are less likely to be found in low-income and ethnic communities [8,9,10], with corner stores, liquor stores, and fast-food restaurants as the more likely options [9,11,12]. Evidently, some food venues target specific populations. For example, studies have noted a higher concentration of fast-food restaurants and convenience stores near schools, where children can be enticed by food and beverage products [13,14,15,16]. 

One critique of existing food environment studies is that most have focused on high-income countries such as the U.S., whereas only a few studies have assessed low- and middle-income countries [17,18,19,20,21,22]. Supermarkets, grocery stores, and convenience stores as well as table-service and fast-food restaurants are the traditional food venues in countries similar to the U.S. However, these food venues may not be present or culturally relevant in low- and middle-income countries [23]. In addition to the aforementioned venues of corner stores, liquor stores, and fast-food restaurants, residents of low- and middle-income countries tend to purchase food and beverages from small venues selling fruit, meat, or fish; indoor and street markets; and street food stands (SFS) [24]. However, very few studies have formally assessed the characteristics (e.g., the types of foods and beverages) of culturally relevant food venues, including SFS. 

Street foods are defined as ready-to-eat foods and beverages sold on the streets by vendors who cook, transport, and display these items in a variety of ways, including in pushcarts, modified bicycles, tricycles and wheelbarrows, buckets, balance poles, and stationary stalls or ships [25,26]. SFS degrees of mobility can range from highly mobile to stationary. One important element of SFS, which is emphasized in the definition, is that they are not permanently fixed—even stationary SFS can easily be moved [27]. Usually, street foods are cooked onsite at the SFS, but sometimes, they are prepared at home and transported to selling points where customers are likely to be found. Street foods can also include highly processed, prepackaged foods such as chips and candy.

In most places, SFS are part of the informal economy, meaning that the vendors do not pay city fees to operate on the streets and are not regulated by city officials [27,28,29,30]. The nature of these casual arrangements can precipitate conflicts with city officials and formal business owners, who may claim that the SFS vendors have unfair advantages and steal customers [29,31]. On some occasions, street food vendors have been harassed by authorities and even forcefully removed from the streets in city-wide cleanses [27,28,32]. Nevertheless, SFS are an urban necessity: they enable many inhabitants of the city to meet their dietary needs. 

SFS offer affordable food and beverage options and represent food security for millions of individuals and families around the world [27,29,31,33,34]. This is especially the case for families and individuals in low-income groups who commute long distances to work and do not return home for meals and cannot afford food from more expensive venues, such as restaurants [27,29,31,33,34,35]. SFS are also a source of income for millions of workers, as they provide opportunities to be self-employed and self-sufficient. These workers include individuals who have fewer opportunities to access formal government or private jobs due to limited formal education or social biases (e.g., gender discrimination) [27,28,29,34,35,36,37,38]. More recently, however, even individuals with higher education are starting to view street-food vending as an entrepreneurial opportunity, as this activity can often generate more income than working in a government position [27,36].

The number of SFS studies has grown in the last decade, but most of these have focused on only two continents: Africa and Asia [33,38,39,40,41,42,43,44]. Outside these areas, research on SFS has been quite limited, despite the fact that some Latin American countries (including Mexico) have a rich history of SFS dating back to pre-colonial times [29]. The few studies on SFS in Mexico have primarily focused on food safety and food contamination [45,46,47,48,49,50,51,52,53,54,55]. Although findings from these studies can help prevent food-borne diseases and inform street-food vendors and consumers about sanitation and food-handling practices, we need studies that can shed light on other aspects of SFS. For example, we need a better understanding of the populations targeted by SFS vendors and the roles that SFS play in food availability.

In Mexico, SFS are a popular source of food and beverages in communities, and they are an integral aspect of the food environment, especially in areas where other venues—such as supermarkets and restaurants—are limited. In a nationally representative food intake survey assessing food expenditure and food consumption away from home, approximately 19% of respondents reported consuming a meal at a restaurant at least once a month, whereas 60% reported consuming a meal, snack, or beverage from SFS at least once a month [50]. Thus, evidence suggests that SFS are a popular source of food consumed away from home, but the evidence is lacking on the groups (e.g., low-, middle- or high-income) targeted by SFS and on the numbers of SFS operating near specific points of access (e.g., schools, homes, and worksites). There is a need for more research, as a lack of understanding of the role of SFS in exposing communities to unhealthy food could lead to negative health outcomes. Addressing this issue is particularly important in Mexico, where a large percentage of the population is either overweight, obese, or suffers from type 2 diabetes [56,57,58].

A challenge in studying SFS is that their availability, density, and distribution may be influenced by changes in social, economic, and political structures and processes. For example, changes in societal perceptions or the state of the economy can lead to changes in the availability, density, and distribution of SFS depending on whether SFS are perceived as beneficial to the community and the local economy [29,59,60]. A clear example of these processes is the recent decriminalization of SFS in the city of Los Angeles, California where SFS were limited to specific areas of the city, but are now allowed to be in almost any part of the city [61]. To our knowledge, no study to date has assessed the availability, density, and distribution of SFS in Mexico City using reliable and validated methods. Most studies have focused on selecting a convenience sample of SFS without fully discussing their numbers, types, and locations. Therefore, the objective of this study is to document the availability, distribution, and density of SFS by neighborhood income level in Mexico City and to identify populations targeted by vendors via specific points of access. 

## 2. Materials and Methods

Data in this observational study were collected through ethnographic fieldwork and direct observations of Mexico City street segments between May and August 2018. Mexico City is the largest and most populated city in Mexico, and it attracts migrants from all over the country, who bring their culinary traditions with them from their home regions. Thus, the city has a rich history of SFS offering foods from a wide array of regions in Mexico [29]. This study included only SFS that met the criteria of the UN’s street food definition: ready-to-eat foods and beverages that are prepared and sold on the streets by vendors using facilities such as mobile, semi-stationary, and stationary stands [25,26]. As such, SFS were excluded from the study if (1) stands were part of establishments with four permanent walls; (2) stands were part of a store or an extension of a vendor’s home, and (3) vendors sold nonfood items or raw foods meant to be prepared and consumed at home. SFS were categorized according to the main type of food or dish they sold. The categories of stands were as follows: cooked meals, snacks, fruits/vegetables, and “other.” Cooked meal stands (e.g., those selling tacos, tortas, tamales, hamburgers, and pizza) mostly offered street foods prepared on the streets, but some foods were prepared at home and taken to selling points where customers were likely to be found. Foods sold at cooked meal stands could be sources of protein and vitamins [29,62,63]. Snack stands (e.g., those selling candy, ice cream, chips, and salted dried seeds) sold highly processed, prepackaged foods, whereas fruit/vegetable stands offered minimally processed foods, such as pieces of raw fruit (e.g., mango) or vegetables (e.g., corn) that could be prepared for consumption onsite. The “other” stands were SFS that sold individual food items that did not fall under any other category (e.g., stands selling coffee, traditional Mexican beverages, or toasted crickets) or that sold a mixture of the aforementioned items.

Research assistants (RA) from the Universidad Autonoma de Mexico-Ecatepec were trained in mapping and ground-truthing techniques to assist with data collection. The research team used a standalone component of the Street Food Stand Assessment Tool (SFSAT) [64] to record the availability (i.e., the presence), density (i.e., number), and distribution (i.e., location) of SFS in different neighborhoods throughout Mexico City. The study was deemed exempt under federal regulation 45, 46, 101 (b) CFR and by Arizona State University’s Institutional Review Board, as the research team did not collect any human data or personal information from street food vendors or customers.

**Figure 1:**
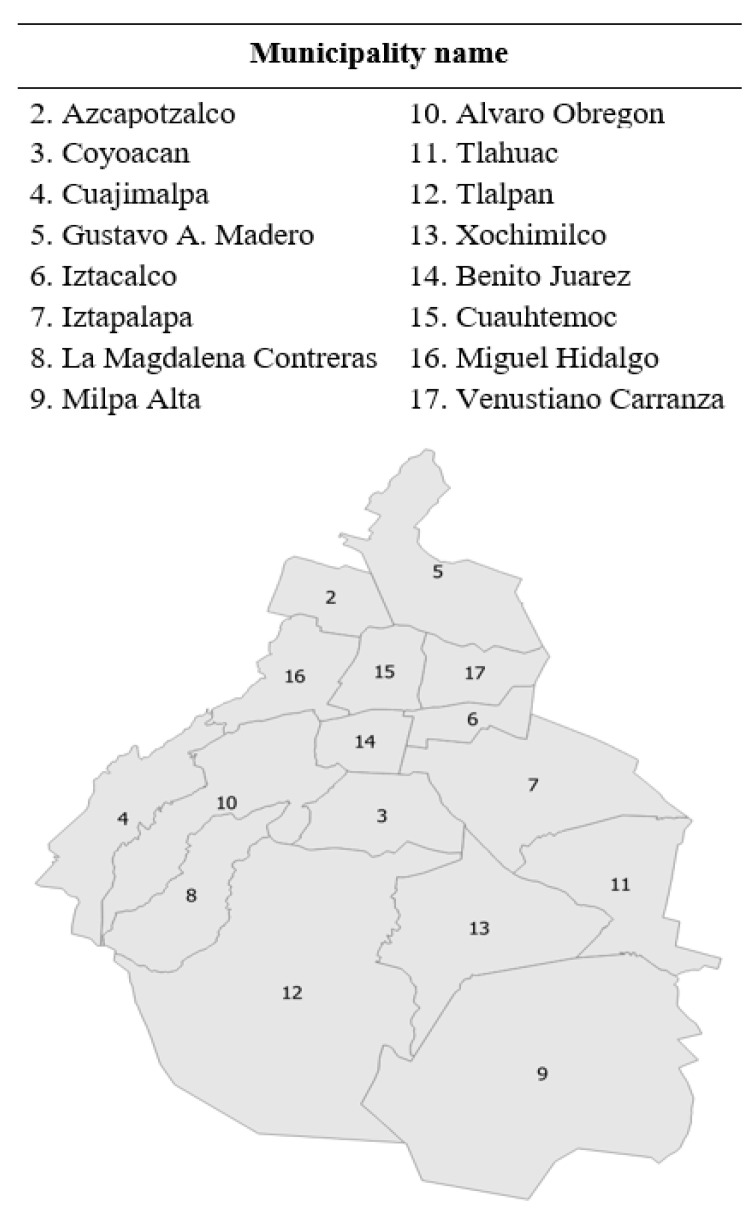
Mexico City’s municipalities.

**Figure 2:**
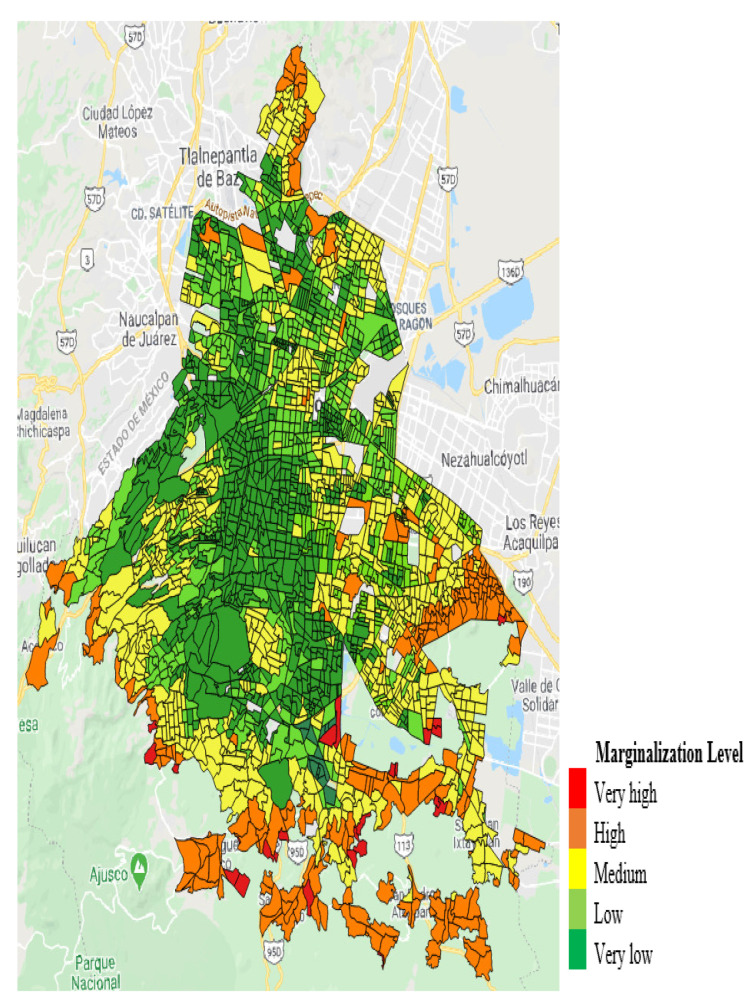
Marginalization levels in Mexico City by census tract.

Mexico City is divided into sixteen municipalities (Figure 1), and these municipalities are further divided into census tracts. The size of the census tracts is dependent on population density (see Table 1). Census tracts are characterized by marginalization levels, which are also referred to as income levels in this paper (Figure 2). These were as follows: very high-, high-, middle-, low-, and very low-marginalization levels representing very low-, low-, middle-, high-, and very high-income levels, respectively [65]. The five income categories were used to select observational areas. However, in the analysis phase, the income levels were merged into three categories: low-income levels (encompassing very high- and high-marginalization levels); middle-income levels (the middle-marginalization level); and high-income levels (encompassing very low- and low-marginalization levels). 

A business directory with information about SFS locations was not readily available due to the informal nature of SFS. Therefore, the research team implemented an alternative strategy to identify a representative sample of SFS across Mexico City. This strategy involved capturing a random sample of street segments to explore the availability, density, and distribution of SFS operating in Mexico City per street segment. First, the census tracts in Mexico City were stratified by income level, and a random sample of four census tracts per income level was selected. A 400-m observational area was drawn around the center of each selected census track using open geographic information system methods to create observational areas [66]. Previous studies have shown that a 400-meter observational area can capture multiple features of a neighborhood, including points of access that street food vendors may be targeting (e.g., schools, parks, worksites, and transportation centers) [67,68]. In addition, a 400-m buffer can capture more than one census tract depending on the size of the tract, the center of which acts as the centroid of the observational area (which is also referred to as the neighborhood).

**Figure 3:**
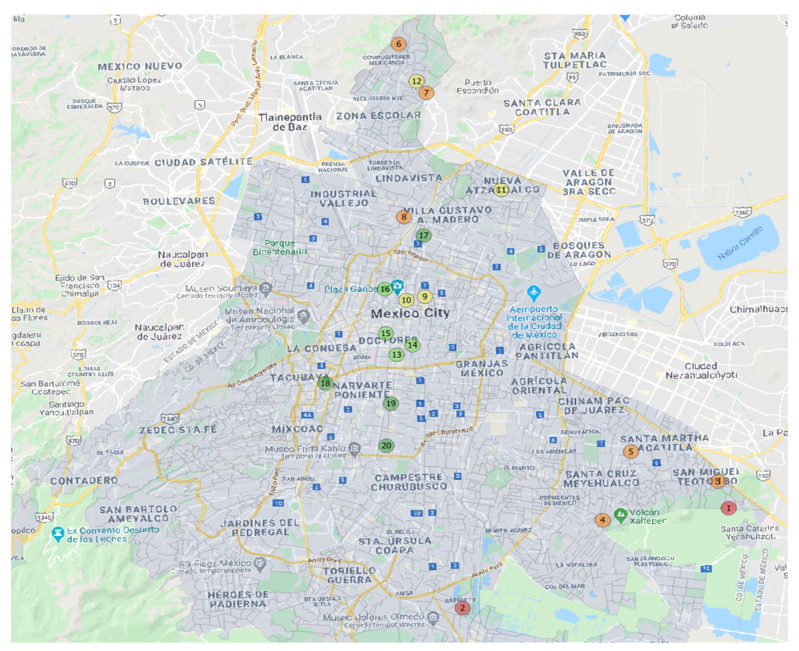
Selected Observational Areas. Note: Red and orange = low-income neighborhoods; yellow = middle-income neighborhoods; green = high-income neighborhoods.

Once the neighborhoods were drawn, all the street segments within each neighborhood were mapped. A street segment was defined as a part of a street intersected by two cross streets or by a cross street on one side and a dead end on the other [69]. Street segments were then subdivided into residential and arterial street segments. A random sample of residential street segments (25%) plus all the arterial street segments was selected for observation [70]. The selected street segments were randomly assigned to morning (8:00–11:59 am), afternoon (12:00–4:59 pm), or evening (5:00–8:59 pm) assessment times to document the SFS variability throughout the day. The research team selected twenty observational areas representing different neighborhoods across Mexico City (Figure 3).

Assessments were completed during weekdays to control for weekend events that could attract vendors to certain areas of the city, which could alter the regular patterns of SFS. Research assistants (RAs) worked in teams of two, and the teams were assigned to assess a random group of street segments. Copies of the neighborhood maps with the selected residential and arterial street segments were given to each team. The RAs conducted street-by-street assessments: they walked the length of the street segments within each neighborhood and assigned a unique identifier to each identified SFS. Subsequently, they documented the following information: the type and total SFS found per SFS category; the points of access within 100 meters of the SFS vending sites; and the geographic locations of the SFS. The information was captured using a standalone component of the SFSAT. The teams were trained to record any instances of and reasons for a street segment not being evaluated (e.g., it was a private or missing street, safety issues, etc.).

SFS availability was defined in this study as the physical presence of any of the four kinds of SFS: cooked meal, snack, fruit/vegetable, or “other” stands. The SFS availability was measured by the following question: “Is the ____ type of stand present on the street segment?” This question appeared four times (i.e., one question for each of the four SFS categories) for each street segment. The RAs selected “yes” if there was at least one SFS of that type present on the street segment and “no” otherwise. 

SFS density was defined in this study as the average number of SFS across street segments within each income level. Density was calculated by dividing the total number of SFS in a category by the total number of street segments assessed in each income level, based on the RAs’ responses to the following question: “How many _____ stands are there on the street segment?” For each street segment, this question appeared four times: one per each SFS category.

SFS distribution was defined in this study as the arrangement of SFS near points of access to certain populations, which may have been the targets of SFS vendors. Point of access included major venues or institutions. The distribution was measured based on the RAs responses to the following question for each SFS on a street segment: “Is the _____ SFS located within 100 m of ___?” The options for points of access included home, sports facility, public transportation center, food inn (mom and pop restaurant also known as fondas), school, church, worksite, park, mall, and restaurant. RAs could select multiple points of access for each type of SFS on a street segment. The points of access were treated in this study as a proxy for the populations potentially targeted by the SFS vendors. 

Descriptive statistics (frequencies) were used to summarize the following neighborhood characteristics: the percentages of segments containing SFS in a particular category; the type of street segments where the SFS were found; and the points of access located within 100 m of the SFS. It is important to highlight that a street segment could contain more than one type of SFS and that the SFS could be found near more than one type of access point. For example, a cooked meal stand and a snack stand could both be present on the same street segment, and those two stands could both be located near a home, a public transportation center, and a worksite. Chi-square tests of independence were performed to examine differences in the SFS availability across neighborhood income levels and in SFS distribution across points of access within 100 m of the SFS by neighborhood income levels. Analyses of variance (ANOVA) were performed to explore differences in the means of the SFS per street segment across neighborhood income levels. Bonferroni adjustments were performed to account for multiple comparisons. Statistical analyses were conducted using Stata statistical software 15 [71]. We hypothesized that there would be higher SFS availability and density in low-income observational areas and areas with heavy pedestrian traffic and high levels of food demand, such as transportation centers, worksites, and schools.

## 3. Results

### 3.1. Descriptive Characteristics

A total of 884 street segments were selected for the assessment (Table 1). However, 13.9% of these segments were not ultimately assessed due to safety or inaccessibility issues (e.g., they were private, uninhabited, or missing street segments). Of the assessed segments (*n* = 761), 36.6% were low-income street segments, 20.4% were middle-income street segments, and 43.0% were high-income street segments. Across all twenty neighborhoods, 66.5% of the assessed segments were residential street segments, and 33.5% were arterial street segments. SFS were present in 27% (*n* = 205) of the assessed street segments. In street segments containing SFS, the teams identified 153 SFS (27%) in low-income segments; 238 SFS (41%) in middle-income segments; and 184 SFS (32%) in high-income segments.

### 3.2. Street Food Stand Availability

Table 2 shows the differences in the SFS category availabilities across neighborhood income levels. Middle-income street segments contained a higher availability of cooked meal stands (27.7%) compared to low-income (10.4%) and high-income (15.0%) street segments (*X*^2^ (2, *N* = 761) = 22.8, *p* < 0.001). Middle income street segments had a higher availability of snack stands (27.1%) compared to low-income (8.24%) and high-income (12.2%) street segments (*X*^2^ (2, *N* = 761) = 30.9, *p* < 0.001). Middle-income street segments also had a high availability of fruit/vegetable stands (10.9%) compared to low-income (6.45%) and high-income (7.95%) street segments, but these differences were not statistically significant (*X*^2^ (2, *N* = 761) = 2.76, *p* = 0.25). Middle-income street segments contained a higher availability of “other” stands (14.2%) compared to low-income (4.66%) and high-income (6.12%) street segments (*X*^2^ (2, *N* = 761) = 14.6, *p* = 0.001).

### 3.3. Street Food Stand Density

The density (average number) of SFS across neighborhood income levels is shown in Table 3. The density of cooked meal stands was higher in middle-income (*M =* 0.63, *SD* = 1.35) compared to low- (*M* = 0.20, *SD* = 0.83) and high-income street segments (*M* = 0.23, *SD* = 0.62; *F* (2,759) = 13.4, *p* < 0.001). In the snack category, there was a higher density of SFS in middle-income (*M* = 0.43, *SD* = 1.04) compared to low-income (*M* = 0.21, *SD* = 0.94) and high-income street segments (*M* = 0.16, *SD* = 0.51; *F* (2,759) = 6.20, *p* < 0.01). The density of fruit/vegetable stands was also high in middle-income (*M* = 0.14, *SD* = 0.43) compared to low-income (*M* = 0.08, *SD* = 0.32) and high-income street segments (*M* = 0.09, *SD* = 0.31), but these differences were not statistically significant, *p* > 0.05. The “other” stands had a higher density in middle-income (*M* = 0.34, *SD* = 1.72) compared to low-income (*M* = 0.06, *SD* = 0.32) and high-income street segments (*M* = 0.09, *SD* = 0.40; *F* (2,759) = 5.95, *p* < 0.01).

### 3.4. Street Food Stand Distribution

Table 4 examines the distribution of SFS across points of access located within 100 m of SFS and neighborhood income levels. Most stands were located near homes (86%), public transportation centers (58%), and worksites (31%). Among cooked meal stands near food inn restaurants, a higher availability of stands was also observed in high-income (49.0%) compared to low-income (24.5%) and middle-income (26.5%) street segments (*X*^2^ (2, *N* = 129) = 15.1, *p* = 0.001). Among cooked meal stands found near worksites, there was a higher availability in high-income (56.9%) compared to low-income (12.3%) or middle-income (30.8%) street segments (*X*^2^ (2, *N* = 145) = 6.24, *p* = 0.04), but these differences were not statistically significant after adjusting for multiple comparisons (*p* > 0.05). In contrast, among snack stands found near public transportation centers, there was a higher availability in low-income (41.1%) compared to middle-income (37.0%) and high-income (21.9%) street segments (*X*^2^ (2, *N* = 273) = 5.98, *p* = 0.05), but these differences were not statistically significant after adjusting for multiple comparisons (*p* > 0.05). There was also a higher availability of snack stands near food inn restaurants in middle-income (44.1%) compared to low-income (41.2%) and high-income (14.7%) street segments (*X*^2^ (2, *N* = 273) = 5.98, *p* = 0.05). Snack stands were also found near worksites, but unlike the cooked meal stands, they exhibited a higher availability in middle-income (38.9%) compared to low-income (33.3%) and high-income (27.8%) street segments (*X*^2^ (2, *N* = 145) = 11.5, *p* = 0.003). There were no statistically significant differences in SFS availability near homes, sports facilities, schools, churches, recreational parks, and shopping centers across neighborhood income levels. 

## 4. Discussion

The purpose of this study was to explore differences in the availability, density, and distribution of SFS across neighborhood income levels in Mexico City. The availability of SFS was high in middle-income neighborhoods compared to low-income and high-income neighborhoods. These differences were consistent for the four SFS categories. Similarly, SFS density was consistently higher in middle-income neighborhoods across all four SFS categories. SFS in all four categories were consistently found near homes, transportation centers, and worksites; at least 8% of SFS were found near these points of access. However, the distribution of SFS near these points of access varied across neighborhoods. For example, fruit/vegetable stands were found near homes in middle-income neighborhoods but near worksites in high-income neighborhoods. Differences in the availability, density, and distribution of SFS categories across neighborhoods could signify differences in the types of food and beverage exposure among customers in different income groups. Additional studies are needed to document the food and beverage availability at SFS and whether differences persist across neighborhoods.

Contrary to our hypothesis, low-income neighborhoods did not exhibit the highest availability of SFS. Middle-income neighborhoods exhibited the highest availability across the four types of SFS categories. We observed a low availability of cooked meals and fruit/vegetable stands particularly in low-income neighborhoods. This finding suggests that low-income communities in Mexico City may be in a vulnerable position, given that street food provides individuals with limited resources with an affordable source of calories and nutrients [72,73,74,75]. In other words, without adequate SFS in their communities, individuals from low-income groups may not have access to essential nutrients. More research is needed to better understand the roles played by SFS in the diets of those from different income groups in Mexico and especially for those in the low-income groups.

While SFS availability assessed whether SFS were present on a street segment, SFS density assessed the numbers and types of SFS present on the street segments. This study found that low-income neighborhoods exhibited the lowest density of cooked meal, fruit/vegetable, and “other” stands, with higher densities of these stands found in middle- and high-income neighborhoods. In this study, the presence of SFS across all three neighborhood income levels suggests that SFS are sources of food for individuals from all income groups in Mexico City. Studies of other low- and middle-income countries have also found a high availability of SFS outside of low-income communities, indicating that individuals from varying economic backgrounds consume street food [28,33,35,76]. However, it is important to note that the presence of SFS in a community does not suggest that residents of that community are the ones mainly consuming street food. It may be that the higher demand for street food in middle- and high-income neighborhoods are due to people from low-income groups congregating in these neighborhoods for work and other activities. Future research is needed to assess the socioeconomic backgrounds of SFS customers and the distances they travel to consume street food.

The research team was interested in exploring the distribution of SFS within 100 m of different points of access to better understand which populations the vendors may have been targeting. All the points of access had nearby SFS. However, differences in SFS distribution across neighborhood income levels were observed for the following SFS categories and points of access: snack stands near food inns, worksites, and transportation centers; and cooked meal stands near food inns, and worksites. However, statistically significant differences across neighborhoods for snack stand stands near transportation centers, and for cooked meals stands near worksites disappeared after adjusting for multiple comparisons. The high distribution of stands near transportation centers and worksites may be explained by the high number of commuters traveling through Mexico City. It is estimated that during working hours, the population in Mexico City swells to over 25 million people [77]. On weekdays, thousands of people from nearby cities and towns use the public transportation system to commute to work in Mexico City, and some spend up to six hours a day during these commutes [78]. Other studies have also found that transportation centers and worksites tend to draw SFS [28,33,79,80]. Individuals who commute long distances or who do not have the time to prepare food at home probably consume most of their food outside their homes. For these people, SFS can act as mobile food sources that can be conveniently located to facilitate access.

The distribution results showed that a small proportion of SFS were found near food inns. Studies have mentioned the proximity of SFS to restaurants when discussing conflicts between formal business owners (i.e., restaurant owners) and SFS vendors regarding the unfair advantages of vendors, who may not pay city, service, or permit fees [29,31]. The proximity of SFS to restaurants may also be associated with the cost of food. In Mexico, the cost of a restaurant meal ranges from 100–500 pesos (equivalent to approximately $5–25 USD) [81,82] whereas the price of a meal at an SFS ranges from 20–100 pesos (equivalent to $1–5 USD). Thus, the cost of restaurant food may be prohibitive for individuals from low and middle-income groups, and SFS may provide more affordable options for these individuals. Future research is needed to better understand restaurant and SFS co-availability, including the products and prices of products at both types of venues, especially when they are adjacent. Studying customer perceptions and food preferences in relation to the adjacent venues could also yield meaningful insights.

The study presented in this manuscript has research and public health implications. Given that SFS are part of an informal business and it is difficult to have an accurate record of how many stands there are and their location at any given time, future studies of the food environment and food availability could benefit from the methods used in this study to identify and recruit random samples of SFS across neighborhoods. These methods can capture samples of stationary, semi-mobile, and highly mobile SFS. While documenting the type of foods available at the food stands and their nutritional value is beyond the scope of this study, the research team documented the presence of a variety of SFS characterized by the type of food and beverage items sold. There was high availability of cooked meal stands. Many of these stands offered traditional Mexican food items based on corn, beans, and other vegetables that are minimally processed and are considered nutritious [61]. However, many other stands were snack stands. The snack stands are characterized by selling highly processed food and beverages such as chips, candy, and sugar-sweetened beverages [61]. The presence of these types of items is a public health concern, because the consumption of highly processed foods and beverages has been associated with negative health outcomes such as obesity, type 2 diabetes, and cardiovascular diseases [83,84,85]. It should be in the interest of community health to identify and encourage the availability of specific SFS (i.e., cooked meals) over others (i.e., snack stands). Some studies have shown that when the availability of unhealthy food items is modified and healthy food items are made available and affordable, people are more likely to select healthier options [86,87,88]. Nevertheless, additional studies are needed to further understand the role SFS may play in dietary intake and health outcomes among the Mexican population.

The state of the economy and local policies can encourage or hinder the presence and availability of SFS. Crashes in the economy have been associated with increases in SFS. For example, New York City has seen a recent increase in the number of unregistered SFS as the result of thousands of people losing their jobs due to the COVID-19 pandemic [59]. Although New York City has a formalized street vending license process, the number of licenses available has been limited. Therefore, many of the new vendors may have not had permits, and when caught without a license they faced expensive violation citations. New York City responded to this crisis by increasing the number of permits available for street vending [60]. These economic and local policy processes have, to some extent, encouraged the availability of SFS. In places like Mexico City, SFS are primarily part of an informal economy. As such, many stands go unregulated by any official government body [29]. The local government pays a blind eye to the presence of SFS, because, in dense urban centers like Mexico City, SFS are seen as an urban necessity: they are a source of affordable and convenient food, as well as a source of income for thousands of people [29,34,39]. However, many SFS policies have been created to remove and eradicate SFS [28,29]. Some of these policies have been encouraged by local business owners for whom SFS represent a source of unfair competition [29,31,32]. On other occasions, SFS have been removed to make cities appealing to tourism [32]. These types of policies are detrimental for community members, especially for those who depend on street food vending as a source of food security and income. Instead of complete removal from the streets, local governments, street food vendors, business owners, and community members should work together to identify ways that can protect the presence of SFS while addressing unfair advantage concerns by competitive business owners.

One of the strengths of this study is its unique and comprehensive approach in using geographic information system methods and direct observations of street segments to identify a representative sample of SFS and to explore the availability, density, and distribution of SFS across neighborhood income levels. Other food environment studies have relied on business directories to identify the locations of food venues [23,89,90,91]. However, this approach would not have been suitable for SFS, given that many stands operate informally and thus, are not listed in business directories. The research team captured a representative sample of SFS by selecting a random sample of observational areas and street segments across income levels and points in time and then conducting direct observations of the street segments. In addition, the study captured several points of access, including worksites, schools, public transportation centers, and other busy locations by selecting 400-m observational areas throughout Mexico City. Previous studies of SFS in Mexico have focused on SFS near schools [54,92,93], limiting the scope of study to children and schools. Our study’s approach is more representative, as it included multiple points of access, meaning that the findings can be generalized to various populations within Mexico City. However, even though we selected twenty observational areas across income levels, our findings cannot be generalized to other Mexican cities; this is one limitation of our study. As Mexico’s capital and economic heart, Mexico City is unusual in that it is highly urbanized and is the country’s most densely populated city. Thus, our findings should not be generalized to less urbanized and less densely populated cities in Mexico. An additional limitation of this study is seasonality. Data collection took place from May to August, which is summertime in Mexico when some workers and their families take vacations. Thus, the SFS availability, density, and distribution may be different during the summer than during other times of the year. Therefore, our findings may not be generalizable to other times of the year.

## 5. Conclusions

This study explored differences in the availability, density, and distribution of SFS across income levels in Mexico City. While SFS were found on low-income street segments, there were higher availability and density of SFS in middle- and high-income street segments. These findings suggest that SFS may be a source of food for people from different economic backgrounds. In addition, the high availability and density of cooked meal stands suggest that SFS may also be a source of healthy food. Future studies are needed to better understand the types of food and beverages sold at SFS and the nutritional value of these items. 

## Figures and Tables

**Table 1:** Street Segment Characteristics Across Neighborhood Income Levels.

		Neighborhood Income Levels		
Street Segment Characteristics (*n* = 761)	Number of Street Segments	Low	Middle	High
	n (%)		n (%)	
**Publicly accessible**				
Yes	761 (86.1)	279 (78.6)	155 (93.4)	327 (90.1)
No	123 (13.9)	76 (21.4)	11 (6.63)	36 (9.92)
**Type**				
Residential	506 (66.5)	240 (86.0)	101 (65.2)	165 (50.5)
Arterial	255 (33.5)	39 (14.0)	54 (34.8)	162 (49.5)
**Observation time**				
Morning	303 (39.8)	133 (47.7)	49 (31.6)	121 (37.1)
Afternoon	270 (35.5)	78 (27.9)	70 (45.2)	122 (37.4)
Evening	188 (24.7)	68 (24.4)	36 (23.2)	84 (25.5)
**SFS found on segment**				
SFS found	205 (26.9)	53 (19.0)	67 (43.2)	85 (26.0)
SFS not found	556 (73.1)	226 (81.0)	88 (56.8)	242 (74.0)
**Street segments not assessed (*n* = 123)**				
Observation time				
Morning	9 (7.32)	7 (9.21)	2 (18.2)	0 (0.00)
Afternoon	30 (24.4)	19 (25.0)	3 (27.3)	5 (13.9)
Evening	84 (68.3)	50 (65.8)	6 (54.5)	31 (86.1)

**Table 2:** Availability SFS Across Neighborhood Income Levels.

	Street Segments Containing SFS (%)				
Type of SFS	Low-Income (*n* = 279)	Middle-Income (*n* = 155)	High-Income (*n* = 327)	X^2^ (df)	*p*-Value
		n (%)			
Cooked meals	29 (10.4)	43 (27.7)	49 (15.0)	22.8 (2)	<0.001 ^a,b^
Snacks	23 (8.24)	42 (27.1)	40 (12.2)	30.9 (2)	<0.001 ^a,b^
Fruits/vegetables	18 (6.45)	17 (10.9)	26 (7.95)	2.76 (2)	0.25
Other	13 (4.66)	22 (14.2)	20 (6.12)	14.6 (2)	<0.001 ^a,b^

Note: a = higher availability in middle- than in low-income neighborhoods; b = higher availability in middle-than high-income neighborhoods.

**Table 3:** SFS Density Across Neighborhood Income Levels.

	Neighborhood Income Level				
Type of SFS	Low-Income	Middle-Income	High-Income	F(df)	*p*-Value
	Mean (SD)				
Cooked meals	0.20 (0.83)	0.63 (1.35)	0.23 (0.62)	13.4 (2759)	<0.001 ^a,b^
Snacks	0.21 (0.94)	0.43 (1.04)	0.16 (0.51)	6.20 (2759)	<0.01 ^a,b^
Fruits/vegetables	0.08 (0.32)	0.14 (0.43)	0.09 (0.31)	1.91 (2759)	0.15
Other	0.06 (0.32)	0.34 (1.72)	0.09 (0.40)	5.95 (2759)	<0.01 ^a,b^

Note: a = higher density in middle- than in low-income neighborhoods; b = higher density in middle- than high-income neighborhoods.

**Table 4:** SFS Distribution by Point of Access Across Neighborhood Income Levels.

		Neighborhood Income Levels				
Type of SFS	Point of Access	Low	Medium	High	X^2^ (df)	*p*-Value
		n (%)				
**Cooked meals**						
	Homes (*n* = 163)	33 (20.2)	69 (42.3)	61 (37.4)	3.44 (2)	0.18
	Sports Facilities (*n* = 23)	3 (13.0)	10 (43.5)	10 (43.5)	1.77 (2)	0.41
	Transportation Centers (*n* = 110)	29 (26.4)	44 (40.0)	37 (33.6)	1.96 (2)	0.37
	Food Inns (*n* = 49)	12 (24.5)	13 (26.5)	24 (49.0)	15.1 (2)	0.001 ^a,b^
	Schools (*n* = 37)	15 (40.5)	10 (27.0)	12 (32.4)	1.80 (2)	0.41
	Churches (*n* = 28)	9 (32.1)	11 (39.3)	8 (28.6)	0.35 (2)	0.84
	Worksites (*n* = 65)	8 (12.3)	20 (30.8)	37 (56.9)	6.24 (2)	0.04 *
	Parks (*n* = 26)	10 (38.5)	8 (30.8)	8 (30.8)	0.16 (2)	0.92
	Malls (*n* = 15)	0 (0.00)	14 (93.3)	1 (6.67)	-	-
	Restaurants (*n* = 26)	0 (0.00)	6 (23.1)	20 (76.9)	-	-
**Snacks**						
	Homes (*n* = 111)	35 (31.5)	43 (38.7)	33 (29.7)	3.87 (2)	0.14
	Sports Facilities (*n* = 9)	4 (44.4)	1 (11.1)	4 (44.4)	5.78 (2)	0.06
	Transportation Centers (*n* = 73)	30 (41.1)	27 (37.0)	16 (21.9)	5.98 (2)	0.05 *
	Food Inns (*n* = 34)	14 (41.2)	15 (44.1)	5 (14.7)	5.98 (2)	0.05 ^a^
	Schools (*n* = 35)	22 (62.9)	8 (22.9)	5 (14.3)	5.04 (2)	0.08
	Churches (*n* = 28)	11 (39.3)	13 (46.4)	4 (14.3)	3.66 (2)	0.16
	Worksites (*n* = 36)	12 (33.3)	14 (38.9)	10 (27.8)	11.5 (2)	0.003 ^a^
	Parks (*n* = 23)	11 (47.8)	5 (21.7)	7 (30.4)	2.57 (2)	0.28
	Malls (*n* = 11)	0 (0.00)	9 (81.8)	2 (18.2)	-	-
	Restaurants (*n* = 20)	0 (0.00)	8 (40.0)	12 (60.0)	-	-
**FV^1^**						
	Homes (*n* = 75)	21 (28.0)	28 (37.3)	26 (34.7)	0.88 (2)	0.64
	Sports Facilities (*n* = 6)	1 (16.7)	2 (33.3)	3 (50.0)	0.67 (2)	0.72
	Transportation Centers (*n* = 51)	16 (31.4)	18 (35.3)	17 (33.3)	0.64 (2)	0.73
	Food Inns (*n* = 27)	6 (22.2)	14 (51.8)	7 (25.9)	1.45 (2)	0.48
	Schools (*n* = 20)	10 (50.0)	4 (20.0)	6 (30.0)	0.50 (2)	0.78
	Churches (*n* = 19)	4 (21.1)	7 (36.8)	8 (42.1)	3.32 (2)	0.19
	Worksites (n= 32)	4 (12.5)	13 (40.6)	15 (46.9)	0.50 (2)	0.78
	Parks (*n* = 15)	5 (33.3)	5 (33.3)	5 (33.3)	0.01 (2)	0.94
	Malls (*n* = 9)	0 (0.00)	7 (77.8)	2 (22.2)	-	-
	Restaurants (*n* = 15)	0 (0.00)	5 (33.3)	10 (66.7)	-	-
**Other**						
	Homes (*n* = 60)	12 (20.0)	31 (51.7)	17 (28.3)	2.82 (2)	0.24
	Sports Facilities (*n* = 25)	5 (20.0)	15 (60.0)	5 (20.0)	4.91 (2)	0.09
	Transportation Centers (*n* = 39)	8 (20.5)	20 (51.3)	11 (28.2)	2.96 (2)	0.23
	Food Inns (*n* = 19)	5 (26.3)	12 (63.2)	2 (10.5)	5.17 (2)	0.07
	Schools (*n* = 13)	4 (30.8)	5 (38.5)	4 (30.8)	2.05 (2)	0.34
	Churches (*n* = 5)	0 (0.00)	4 (80.0)	1 (20.0)	-	-
	Worksites (*n* = 12)	0 (0.00)	8 (66.7)	4 (33.3)	-	-
	Parks (*n* = 16)	3 (18.8)	9 (56.2)	4 (25.0)	4.86 (2)	0.09
	Malls (*n* = 4)	0 (0.00)	3 (75.0)	1 (25.0)	-	-
	Restaurants (*n* = 5)	0 (0.00)	4 (80.0)	1 (20.0)	-	-

Note FV^1^ = fruits and vegetables. - = calculation not performed due to small sample size. * = no statistically significant differences after adjusting for multiple comparisons. a = higher distribution in high- than in low-income neighborhoods; b = higher distribution in high- than middle-income neighborhoods.

## Data Availability

Data available upon request.
